# Solid-State Lithium Ion Supercapacitor for Voltage Control of Skyrmions

**DOI:** 10.1021/acs.nanolett.2c04731

**Published:** 2023-04-13

**Authors:** Maria Ameziane, Joonatan Huhtasalo, Lukáš Flajšman, Rhodri Mansell, Sebastiaan van Dijken

**Affiliations:** NanoSpin, Department of Applied Physics, Aalto University School of Science, P.O. Box 15100, FI-00076 Aalto, Finland

**Keywords:** skyrmions, magnetoionics, supercapacitor, Li ion migration, neuromorphic computing

## Abstract

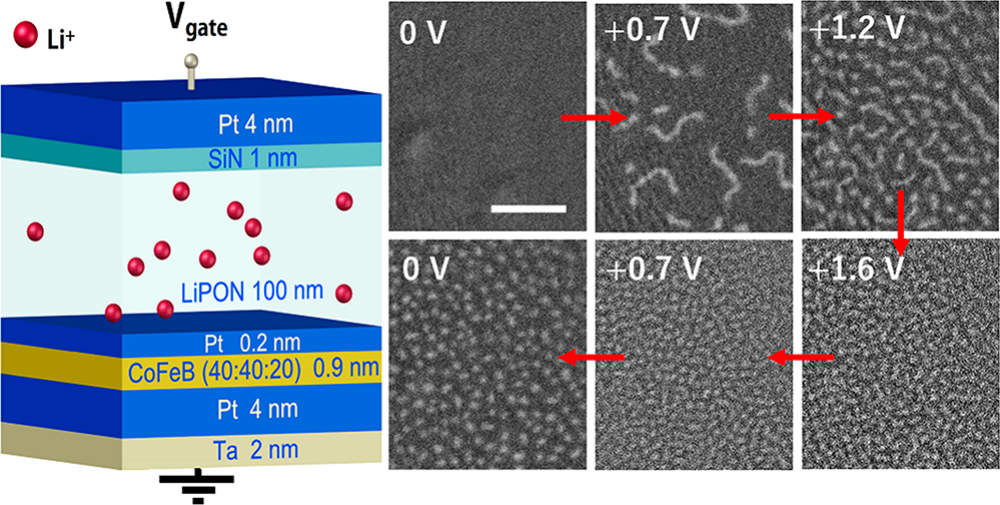

Ionic control of magnetism gives rise to high magnetoelectric coupling efficiencies at low voltages, which is essential for low-power magnetism-based nonconventional computing technologies. However, for on-chip applications, magnetoionic devices typically suffer from slow kinetics, poor cyclability, impractical liquid architectures, or strong ambient effects. As a route to overcoming these problems, we demonstrate a LiPON-based solid-state ionic supercapacitor with a magnetic Pt/Co_40_Fe_40_B_20_/Pt thin-film electrode which enables voltage control of a magnetic skyrmion state. Skyrmion nucleation and annihilation are caused by Li ion accumulation and depletion at the magnetic interface under an applied voltage. The skyrmion density can be controlled through dc applied voltages or through voltage pulses. The skyrmions are nucleated by single 60 μs voltage pulses, and devices are cycled 750000 times without loss of electrical performance. Our results demonstrate a simple and robust approach to ionic control of magnetism in spin-based devices.

Controlling magnetism through applied voltages would allow for the creation of a new class of low-energy nonconventional computing devices. For technological applications, the voltage-induced changes need to be fast as well as reversible and have a strong impact on the magnetic system. The ability to induce large magnetic effects at small voltages has led to an increasing interest in magnetoionic approaches.^1−3^ Previous works have shown that magnetism can be altered ionically through redox reactions,^4−9^ ion intercalation,^10−15^ or the formation of an electronic double layer at solid ion/liquid interfaces.^16,17^ Devices exploiting magnetoionics have been used to control various magnetic properties including the saturation magnetization,^4−8,10−12^ magnetic anisotropy,^4−7,9,16^ and Dzyaloshinskii–Moriya interaction (DMI).^18,19^ The main technological bottleneck for ionically controlled magnetism is the need to apply voltages for extended periods to create sizable effects at room temperature.

Here we take a different approach to ionic control of magnetism by creating a solid-state supercapacitor.^20−22^ The large capacitance of supercapacitors is generated by ion adsorption on the electrodes leading to the creation of an electrical double layer, surface redox reactions, or ion intercalation. Using a Li-enriched LiPON layer as the ion conduction layer, we demonstrate fast, reversible, and durable voltage control of magnetism. In particular, we control magnetic skyrmions—topologically distinct quasiparticles of interest in magnetic data storage and nonconventional computing devices.^23−28^ Previously, voltage control of skyrmions has been shown through interfacial charge modulation,^18,29−32,34^ strain transfer from piezoelectrics,^35,36^ and locally applied electric fields.^37^ However, ionic approaches have been shown to allow greater magnetic modulation effects than directly applied electric fields^6,15^ and avoid the need for the thick crystalline substrates used in strain-transfer devices.^35,36^ By integrating a skyrmion-hosting magnetic thin-film structure with a supercapacitor, we demonstrate nucleation and annihilation of skyrmions through sub-100 μs voltage pulses, a continuously controllable skyrmion density, and the ability to extensively cycle the magnetic state without degradation. The effects demonstrated here are a crucial step toward technological applications, particularly neuromorphic computing,^25−28^ with magnetoionic devices. Such applications require nonlinear effects and short-term memory, both of which are seen here in the dependence of the skyrmion density on voltage. The combination of these effects with the ability to use short voltage pulses and the retention of the properties under significant voltage cycling opens interesting pathways toward useful devices.

As shown in Figure 1a, the magnetron-sputtered structure consists of an ionically conducting, 100 nm thick Li-enriched lithium phosphorus oxynitride (LiPON) layer sandwiched between a 1 nm SiN/4 nm Pt top gate electrode and a 2 nm Ta/4 nm Pt/0.9 nm CoFeB (40:40:20)/0.2 nm Pt bottom electrode. This structure is patterned into 500 μm × 500 μm crossbar junctions shown in Figure 1b,c (see Methods in the Supporting Information). Magnetic hysteresis loops of one junction recorded under an applied bias voltage using polar magneto-optical Kerr effect (MOKE) microscopy are shown in Figure 1d, with corresponding MOKE images depicted in Figure 1e. The voltage is applied for one minute before the loops are taken, which allows the system to reach an equilibrium state. At negative voltage the positively charged Li ions move away from the Pt/CoFeB/Pt electrode, leading to a square hysteresis loop and a fully saturated film magnetization at 0 mT and +0.7 mT. The perpendicular anisotropy is provided by the two Pt interfaces. The upper monolayer thick Pt layer is required to stabilize the perpendicular magnetic state. The zero-voltage state shows a slanted hysteresis loop with magnetic stripe domains at 0 mT and a sparse skyrmion state at +0.7 mT. The application of a positive voltage slants the hysteresis loop further, and it increases the density of the stripe domains (0 mT) and skyrmions (+0.7 mT). At positive voltage the Li ions move toward the magnetic layer. Further details on the determination that the domains are skyrmionic bubbles can be found in the Supporting Information on the extraction of magnetic parameters.

**Figure 1:**
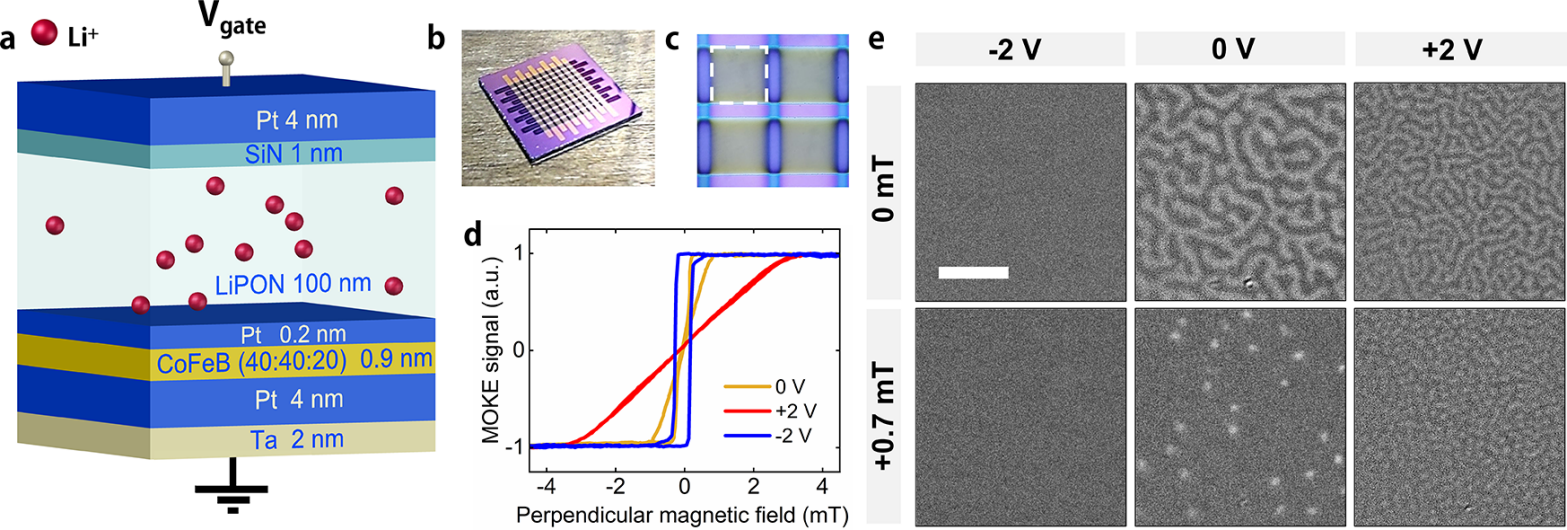
Materials system and voltage control of magnetism. (a) Schematic of the magnetoionic heterostructure. Voltage is applied to the top gate electrode with the bottom electrode grounded. (b) Image of the crossbar sample. Both top and bottom electrodes are 500 μm wide. (c) Close-up image of the device. One junction area is outlined with a dashed white line. (d) Polar MOKE hysteresis loops recorded under 0 V, +2 V, and −2 V bias voltage. (e) MOKE microscopy images for the same bias voltages under 0mT and +0.7 mT perpendicular field. The scale bar indicates 10 μm.

To investigate control of the skyrmion density, the voltage was stepped from −1.0 V to +2.0 V and back to −1.0 V at 0.1 V intervals (Figure 2), with the voltage kept at each level for 20 s before the image is taken. MOKE microscopy images of the CoFeB film at selected gate voltages recorded in +0.7 mT perpendicular field are shown in Figure 2a. Starting from a saturated magnetization state at −1.0 V, inverse stripe domains form at +0.7 V, followed by the nucleation of sparse skyrmions at +0.8 V. The density of the mixed stripe and skyrmion state increases with voltage before morphing into a dense skyrmion lattice at +1.6 V. Hereafter, the skyrmion density increases further up to +2.0 V. Sweeping the voltage in the opposite direction reduces the skyrmion density gradually until all skyrmions are annihilated at −0.6 V. Figure 2b summarizes the skyrmion density during the voltage sweep. The hysteresis demonstrates the existence of a memory effect in the device, enabling access to a continuous range of skyrmion states, which is a requirement for neuromorphic devices. Besides control over skyrmion nucleation and annihilation, the gate voltage also tunes the skyrmion size (Figure 2c). The first skyrmions appearing at +0.8 V are large (∼1.6 μm), but their diameter decreases continuously up to +2.0 V (∼1.2 μm) (see Methods in the Supporting Information). Sweeping the voltage in the negative direction only has a small effect on the skyrmion size. Full reversibility between a reproducible skyrmion state and no skyrmions upon repeated voltage cycling is demonstrated in Figure 2d, where the voltage is held at each step for 1 min before data collection.

**Figure 2:**
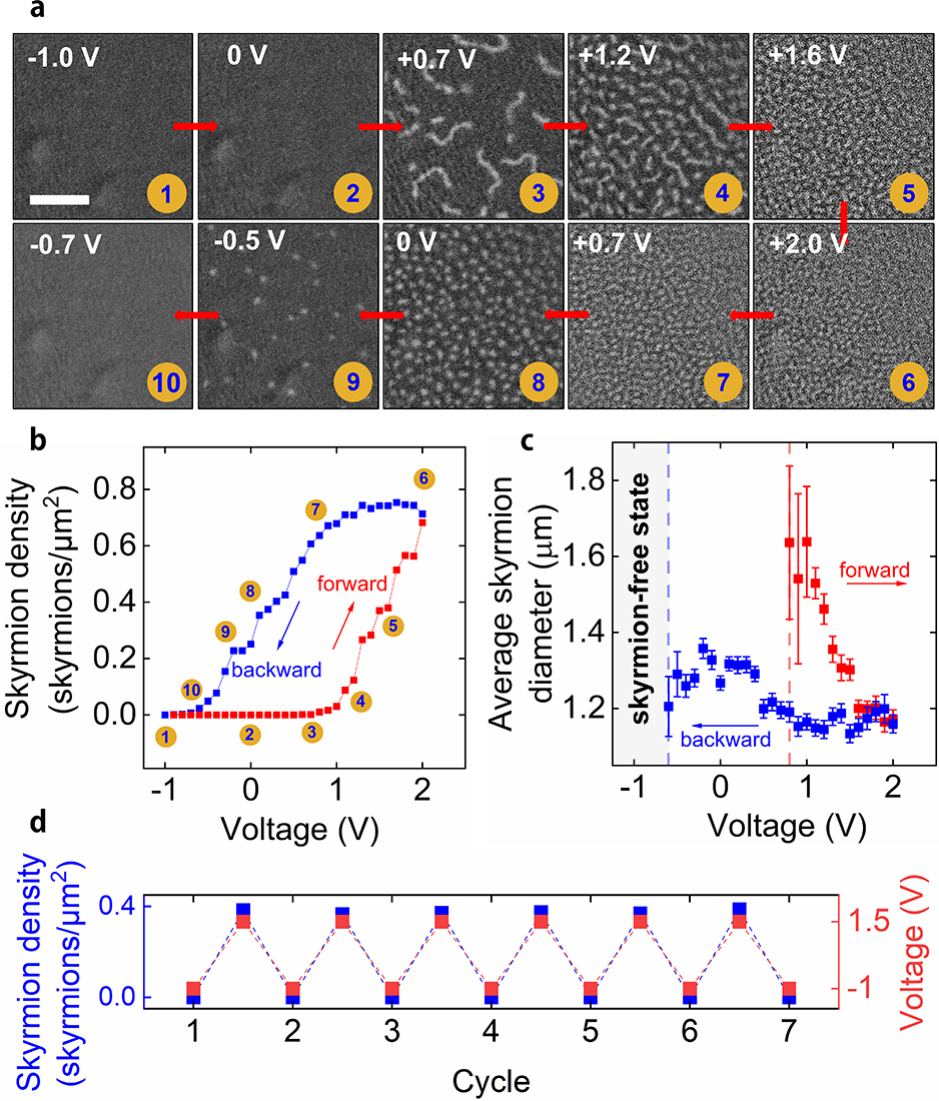
Voltage dependence of the skyrmion state. (a) Polar MOKE microscopy images recorded while sweeping the applied voltage from −1.0 V to +2.0 V and back. The perpendicular magnetic field is +0.7 mT. The scale bar corresponds to 10 μm. (b) Skyrmion density during the voltage sweep. The error in the determination of the density is similar in size to the data points. (c) Average skyrmion diameter during the voltage sweep. The error bars show the standard error of the mean size. (d) Reversible toggling of the skyrmion density by switching the voltage between −1.0 V and +1.5 V. The voltage is applied for 1 min before data collection.

For applications, devices are likely to be controlled by voltage pulses, where the response to both the application and removal of a voltage is relevant to the device operation. To investigate the decay of the skyrmion state over time at zero-bias voltage, we applied +2.0 V for 1 min to a crossbar junction followed by setting the voltage to zero. The skyrmion density as a function of time is shown in Figure 3a, and MOKE microscopy images at various times are depicted in Figure 3b. The decay constant is found to be approximately 8 min.

**Figure 3:**
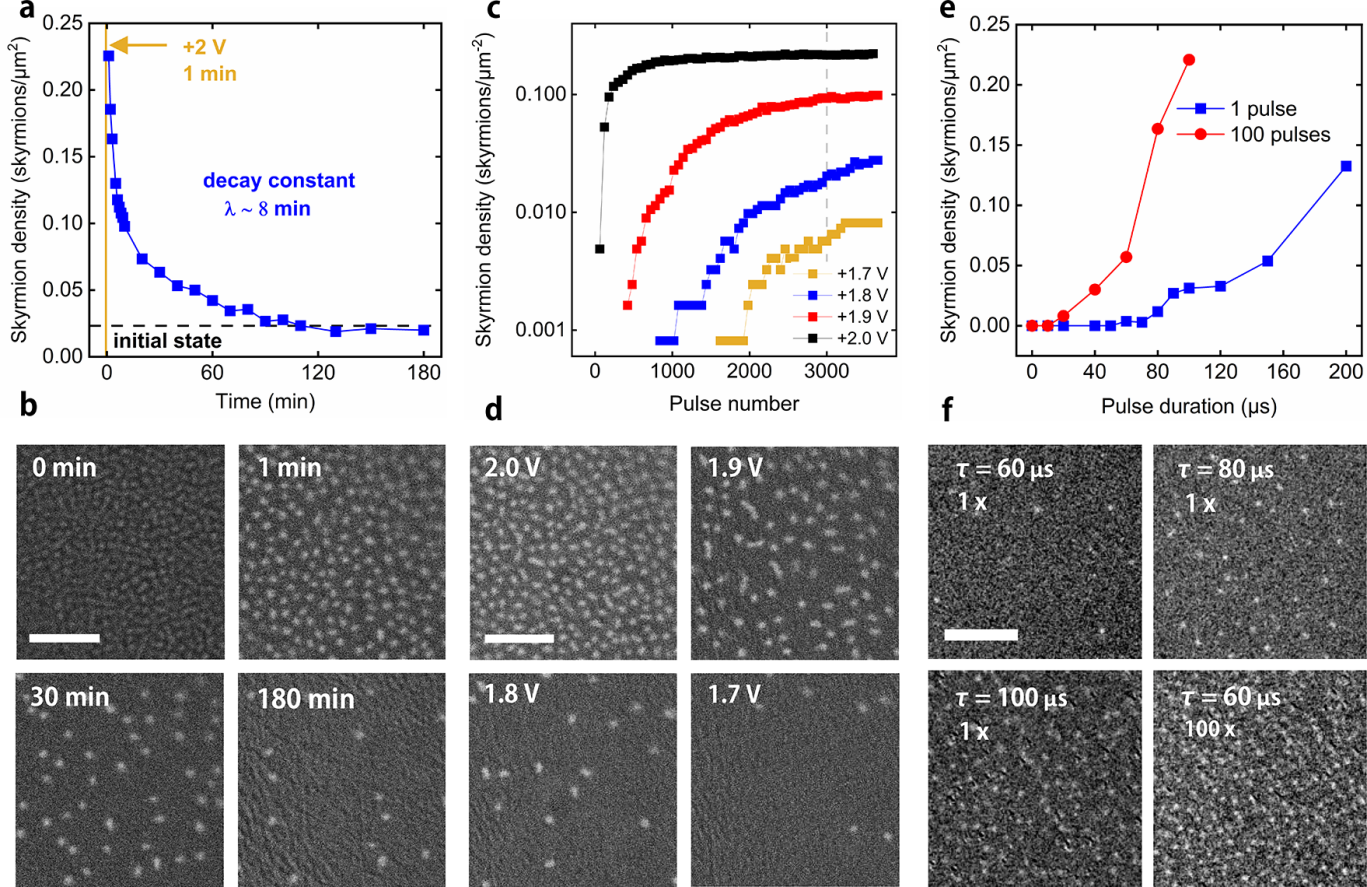
Control of skyrmions by voltage pulse number, pulse amplitude, and pulse duration. (a) Time evolution of the skyrmion density at 0 V after skyrmion nucleation at +2.0 V for 1 min. (b) MOKE microscopy images recorded during the retention experiment shown in (a). The scale bar indicates 10 μm. (c) Skyrmion density as a function of voltage pulse number using a pulse duration of 250 ms with a 50% duty cycle. The pulse amplitude is varied from +1.7 V to +2.0 V. (d) MOKE microscopy images recorded after 3000 pulses for each of the applied voltages. The scale bar indicates 10 μm. (e) Skyrmion density after applying a single voltage pulse and a sequence of 100 voltage pulses to the uniform magnetization state. The amplitude of the pulse is fixed at +10.0 V, and the duration of the pulse is varied. The 100-pulse sequence has a duty cycle of 10%. (f) MOKE microscopy images recorded after 60 μs, 80 μs, and 100 μs for both 1 and 100 pulses. The scale bar corresponds to 20 μm. All experiments used a +0.7 mT perpendicular magnetic field.

To assess the dynamic response of our magnetoionic device under voltage pulsing, we applied 250 ms long voltage pulses with magnitudes ranging from +1.7 V to +2.0 V at 500 ms intervals and monitored the skyrmion density over time (Figure 3c). The device was reset to a skyrmion-free state between each series of pulses by applying −2 V for 5 s. MOKE microscopy images of the CoFeB film taken after 3000 pulses are shown in Figure 3d for four different pulse voltages. Two clear features stand out: first that the rate of approach to an equilibrium value is much faster at higher applied voltage and second that the equilibrium skyrmion density is much higher at higher applied voltage. The device shown here was cycled over 50000 times while retaining the voltage control of the skyrmion state.

We further exploit the dependence of the skyrmion density on voltage to probe the skyrmion nucleation kinetics at shorter time scales. By applying a single pulse of +10 V, we show that the pulse width required for skyrmion nucleation can be as low as 60 μs (Figure 3e, blue curve). In these experiments, the device was reset by applying a –0.8 V gate voltage for 5 s before each voltage pulse, and the skyrmion density was recorded for a few seconds after the pulse. For a sequence of 100 identical pulses the number of nucleated skyrmions increases, and a pulse duration of just 20 μs is already sufficient to nucleate skyrmions (Figure 3e, red curve). MOKE microscopy images of the crossbar junction after a pulse or pulse sequence are presented in Figure 3f for pulse durations between 60 μs and 100 μs.

To understand the functioning of the devices, we turn to electrical characterization. Cyclic voltammograms (CVs) of the supercapacitor structure show a largely rectangular shape with no peaks indicative of redox processes (Figure 4a). As shown in Figure 4b, for low voltage ranges the current at 0 V is a slowly increasing function of the voltage range with the junction current increasing notably for larger voltage ranges. This suggests that both electric double layer and electrochemical mechanisms are present, with the electrochemical mechanism dominating at higher voltages.^8^ Given the material system it is expected that the electrochemical mechanism is intercalation of the Li ions. The capacitance of the junction is calculated to be 0.18 μF at 1 V/s, which is equivalent to a capacity of 72 μF/cm^2^, showing large storage capability typical of supercapacitors. In Figure 4c electrical impedance spectroscopy is shown, giving a steep line at lower frequencies as expected from a capacitance-dominated device. The supercapacitor system is highly cyclable, with Figure 4d showing the CV (at 50 mV/s, giving squarer loops than in Figure 4a) before and after cycling 750000 times with 250 ms pulses at ±2 V. The changes in the CV with cycling are likely due to the formation of reaction products at the electrodes.^21^ This occurs after a relatively small number of cycles (see the black line in Figure 4d), and the changes do not seem to impact the electrical cyclability of the device. The high cyclability of the supercapacitor is related to the lack of a Li ion storage layer which means that there are limited Faradaic changes to the sample, and a relatively small number of Li ions move around the structure as compared to batteries. Moreover, our supercapacitor is intrinsically fast with a characteristic charge/discharge time of 560 μs, derived from measurements of the frequency-dependent capacitance (see Figure S1d). This underlies the ability to use short voltage pulses to control the skyrmions, and the faster response of supercapacitors compared to battery-like structures is a key advantage. The other panels of Figure S1 provide additional information on the electrical properties of the supercapacitor structure, including leakage current, open circuit voltage, and its electrical impedance as a function of frequency.

**Figure 4:**
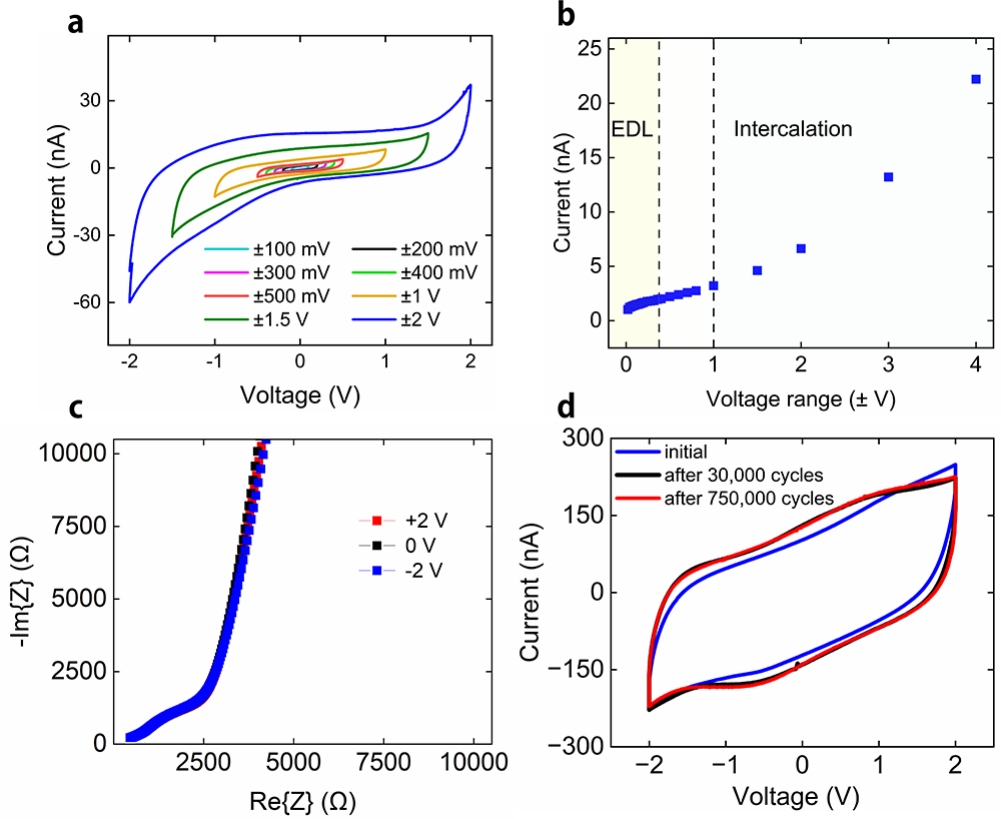
Electrical characterization of supercapacitor junctions. (a) Cyclic voltammograms recorded for different voltage ranges at 10 mV/s scan rate. (b) Junction current at 0 V as a function of voltage range, derived from cyclic voltammetry with a 10 mV/s scan rate. The voltage range is symmetric around 0 V. (c) Electrical impedance spectroscopy measurements on a junction using a 100 mV ac driving voltage with bias voltages of −2 V, 0 V, and +2 V. (d) Cyclic voltammograms at a 50 mV/s scan rate. An initial cyclic voltammogram (blue) was followed by cycling the junction between −2 V and +2 V with a period of 250 ms for 30000 cycles after which a second cyclic voltammogram (black) was recorded and then a third cyclic voltammogram (red) after a total of 750000 cycles.

The combination of magnetic and electrical data shows that the accumulation or depletion of Li ions at the CoFeB/Pt interface causes large changes to the magnetic state at low voltages. Notably, the onset of skyrmion nucleation in Figure 2b occurs around +0.8 V, similar to the voltage at which intercalation starts to dominate (see Figure 4b). Li ions are driven by the applied positive voltage to the LiPON/Pt interface but will further intercalate into the magnetic layer because the upper Pt layer is only a monolayer thick. The density of intercalated ions depends on the interfacial electric field, which itself depends on the applied voltage. From previous research on a similar system,^15^ it is likely that some of the Li diffuses to the bottom CoFeB/Pt interface.

Values for the perpendicular magnetic anisotropy (*K*_u_) and the Dzyaloshinskii–Moriya interaction constant (*D*), along with saturation magnetization (*M*_s_) and exchange constant (*A*_ex_), were estimated from a thin film sample with a similar structure (see Figure S2). *K*_u_ and *D* were found to be 9.96 × 10^5^ J/m^3^ and 0.74 mJ/m^2^, respectively, which is consistent with the creation of bubble-like magnetic skyrmions in this sample at around the sizes seen in Figures 1 and 2 (see section on micromagnetic simulations of the skyrmion energy and Figure S3 in the Supporting Information). From our previous work combining experiments and density functional theory calculations,^15^ the insertion of Li ions at the CoFeB/Pt interface is expected to reduce the perpendicular magnetic anisotropy without reducing the magnetization.^15^ The insertion of Li ions at the CoFeB/Pt interfaces disrupts the orbital hybridization which gives rise to both the perpendicular magnetic anisotropy and the DMI. However, the changes in anisotropy were too small to be measured accurately via MOKE in this sample. Compared to the battery-like device reported in ref (15), the supercapacitor structure studied here supplies a smaller number of Li ions at a given voltage to the magnetic film due to the absence of a separate Li ion storage electrode.

The effect of reducing the magnetic anisotropy is to reduce the energy barrier to skyrmion nucleation and to stabilize skyrmions relative to the uniform state^29^ (see Figure S3). For the positive sweep direction in Figure 2b the data show that there is a simultaneous increase in density and reduction in size of the skyrmions with increasing applied voltage. In the negative sweep direction, the skyrmion diameter increases from +2 V as the voltage is reduced to 0 V and then decreases for negative voltages. For isolated skyrmions, a reduction (increase) in anisotropy is expected to lead to an increase (reduction) in the skyrmion size.^29,38^ However, only the backward direction data from 0 V to −0.5 V seems consistent with that. Instead, a decrease in size is seen with increasing voltage for the forward direction, which could suggest that the DMI is also reduced by the accumulation of Li ions at the CoFeB/Pt interface.^18^ However, other factors can also affect the skyrmion size. Skyrmions at lower densities may preferentially nucleate at defect sites,^39^ with the lower anisotropy at these sites leading to larger skyrmions. The effect of increasing skyrmion density could also cause a reduction in the skyrmion size due to a reduction in the net dipolar field. The distinction between the forward and backward branches is likely also due to the existence of stripe domains in the forward branch, which will also change the dipole fields of the system and so affect the skyrmion size.

The time-dependent experiments give insight into the time scales of the phenomena. The decay time of the skyrmion state in Figure 3a corresponds to an energy barrier of around 0.38 eV, similar to that expected for the thermally activated hopping motion of Li ions within LiPON.^15^ To minimize the internal electric field within the ion conduction layer, there is a thermally activated redistribution of Li ions within the layer, causing the skyrmions to consequently annihilate over time. This also explains the results of the pulsed experiments in Figure 3c. Here the positive voltage pulses cause the accumulation of Li ions at the CoFeB/Pt interface, which decreases the skyrmion nucleation barrier, while during the off state the accumulated ions decay. The concentration of interfacial Li ions increases with the number of voltage pulses until the decay in the off state balances a further increase during the on state. The increasing final density with increasing applied voltage is consistent with the data in Figure 2c and is due to the greater equilibrium concentration of Li in the CoFeB/Pt layers at higher voltages. The process of reaching equilibrium is fast for Li ions due to the high ionic conductivity of LiPON^15,21^ (see also Figure S4).

For the submillisecond pulses used in Figure 3e, there is a further effect. Now the barrier for skyrmion nucleation is lowered rapidly and then increases again as the Li accumulation decays. However, the nucleation of skyrmions occurs on a time scale longer than the voltage pulses, leading to a peak in skyrmion density around a second after the pulse (Figure S4). Therefore, the speed of the devices is also limited by the thermally activated nucleation of the skyrmions.

Fast and durable voltage control of skyrmions in Li ion supercapacitor structures, as shown here, offers attractive pathways to the implementation of neuromorphic devices such as synapse-based neural networks^25^ and reservoir computers.^26−28^ Skyrmions have many degrees of freedom such as position, size, and density which provides a high-dimensional space onto which inputs can be transformed, a key feature in reservoir computing.^27^ Proof of concepts demonstrating the suitability of skyrmion dynamics for neuromorphic computing have thus far utilized magnetic fields or electric currents to control the skyrmion state. Voltage gating of a skyrmion-hosting magnetic film provides good scalability and energy efficiency in combination with deterministic accumulation/dissipation, short-term memory, and nonlinearity. For instance, reversible nucleation and annihilation of skyrmions through the application of positive and negative voltages (Figure 2) enables the emulation of synaptic weight changes during potentiation and depression, while the decay of the skyrmion state after voltage pulsing (Figure 3a,b) provides short-term memory to temporarily store information and triggers outputs based on the time-dependent history of voltage inputs. Nonlinearity of voltage-driven skyrmion dynamics, which is another key requirement for neuromorphic processing, is demonstrated in our supercapacitors by varying the amplitude (Figure 3c,d) and duration (Figure 3e,f) of the voltage pulses. Finally, we note that the complex interplay between the dynamics of Li ion migration in the solid-state LiPON electrolyte and the ensuing nonlinear dynamics of skyrmions in the thin magnetic film offers great flexibility in the design of functional responses and further device optimization.

For applications in neuromorphic computing, nonlinear effects and short-term memory are required, as we show here using voltage control of the skyrmion state. However, useful devices need to show the necessary effects and be both fast and durable. These two aspects have been a particular problem for ionics-based devices. Ion migration tends to be slow, and devices degrade rapidly due to structural changes caused by electrochemical reactions. In this paper, we demonstrate that using a Li ion-based supercapacitor allows the control of skyrmions with both the necessary physical effects for useful devices and that this can be accomplished at relatively high speeds with a high cyclability.

In summary, we have shown that skyrmions in a Pt/CoFeB/Pt thin-film structure can be created and annihilated in a fully voltage-controlled all-solid-state device via reversible Li ion migration at room temperature. The hysteretic behavior of the device with respect to the voltage sweep direction, the nonlinear effects observed as a function of voltage pulse number and pulse duration, and the decay behavior at zero voltage constitute properties suitable for neuromorphic device architectures. The use of a supercapacitor enables skyrmion nucleation with single voltage pulses down to 60 μs, combined with extensive cycling of the junctions. Further downscaling of the device from the 100 nm thick solid-state electrolyte used here may allow access to submicrosecond functionality.

## Supporting Information Available

Methods and additional data, including electrical characterization, measurements to extract magnetic parameters, micromagnetic simulations of the skyrmion energy, and fast voltage pulsing experiments (PDF)
