# Supplementary material for: Solid-State Lithium Ion Supercapacitor for Voltage Control of Skyrmions
Source: Nano Lett. 2023 Apr 13;23(8):3167–73. doi: 10.1021/acs.nanolett.2c04731 (PMC10141402; doi:10.1021/acs.nanolett.2c04731)
Supplement: Supplementary file 1 — nl2c04731_si_001.pdf [file nl2c04731_si_001.pdf]

Solid-state lithium-ion supercapacitor for voltage control of skyrmions

Supporting Information

Maria Ameziane, Joonatan Huhtasalo, Lukáš Flajšman, Rhodri Mansell* and Sebastiaan van Dijken*

NanoSpin, Department of Applied Physics, Aalto University School of Science, P.O. Box 15100, FI-00076 Aalto, Finland

* Corresponding authors: rhodri.mansell@aalto.fi; sebastiaan.van.dijken@aalto.fi

Methods

Sample fabrication

The magneto-ionic supercapacitors were fabricated by shadow-masking in a Kurt J. Lesker magnetron sputtering system with a base pressure of 3.0×10^{-8} mbar. The 2 nm Ta/4 nm Pt/0.9 nm CoFeB (40:40:20)/0.2 nm Pt bottom electrode and 100 nm LiPON electrolyte were first deposited through a shadow mask with 500 μm wide parallel stripes on a 10 mm × 10 mm Si substrate with 300 nm thermal oxide. The LiPON layer was grown by reactive RF sputtering from a Li-enriched lithium phosphate ($Li_{3.3}PO_4$) target under 2 sccm Ar and 20 sccm N_2 flow. To protect the LiPON electrolyte from degrading, we capped it by 1 nm SiN before breaking vacuum. Next, the shadow mask was rotated by 90° and the 4 nm Pt top electrode was sputtered through the mask to create an array of crossbar junctions. The entire heterostructure was grown at room temperature.

Sample characterization

The magnetic properties of the crossbar junctions were characterized using an Evico MOKE microscope equipped with an electromagnet for applying a perpendicular magnetic field. A 50× lens with 0.8 NA was used for imaging with white light, giving a nominal resolution around 350 nm. For the application of voltages, the crossbar junctions were wire-bonded using a 5330 F&S Bondtec system. A Keithley 2450 source meter was used to apply voltages during magnetic characterization and to analyze the electrical properties of the supercapacitors (cyclic voltammograms, open-circuit voltage, leakage current). Electrochemical impedance spectroscopy data were collected using an E4980A

Agilent Precision LCR meter. Vibrating sample magnetometry measurements were carried out with a Quantum Design PPMS system.

Skyrmion analysis

The skyrmion density was determined from the collected MOKE microscopy images using the TrackMate plugin of ImageJ [S1], adjusting the size and threshold parameters. To determine the size of the skyrmions in Figure 2c of the manuscript, a MATLAB script was created where the MOKE images were treated with a Gaussian filter and then binarized. Connected regions due to skyrmions and magnetic stripe domains were then found, and the skyrmions were differentiated from stripe domains based on their size and circularity. The area and equivalent diameter of the selected areas were subsequently determined.

Electrical characterization

Figure S1a presents cyclic voltammograms (CVs) for small voltage ranges. The crossbar junction shows a large capacitive voltage change consistent with the formation of an electrical double layer. As shown in Figure 4a of the main paper, the shape of the CVs changes for larger voltage ranges, suggesting the activation of an electrochemical mechanism, most likely Li ion intercalation. Figure S1b depicts the junction leakage current under ±2.5 V bias voltage. The leakage current is only a few nA, showing the high electrical resistivity of the LiPON layer. Because the heterostructure is mostly symmetric, the open-circuit voltage is small. By setting the current across a crossbar junction to zero and allowing the voltage to reach equilibrium, we measured an open-circuit voltage of 44 mV (Figure S1c). The intrinsic charge/discharge time of the supercapacitor is 560 μs, as determined from the peak in the imaginary part of the capacitance plotted against frequency (Figure S1d). This is about one order of magnitude slower than the time needed to nucleate skyrmions (see Figure 3e,f in the main paper). Improving the intrinsic charge/discharge time is likely a good way to further speed up the voltage-induced skyrmion nucleation process.

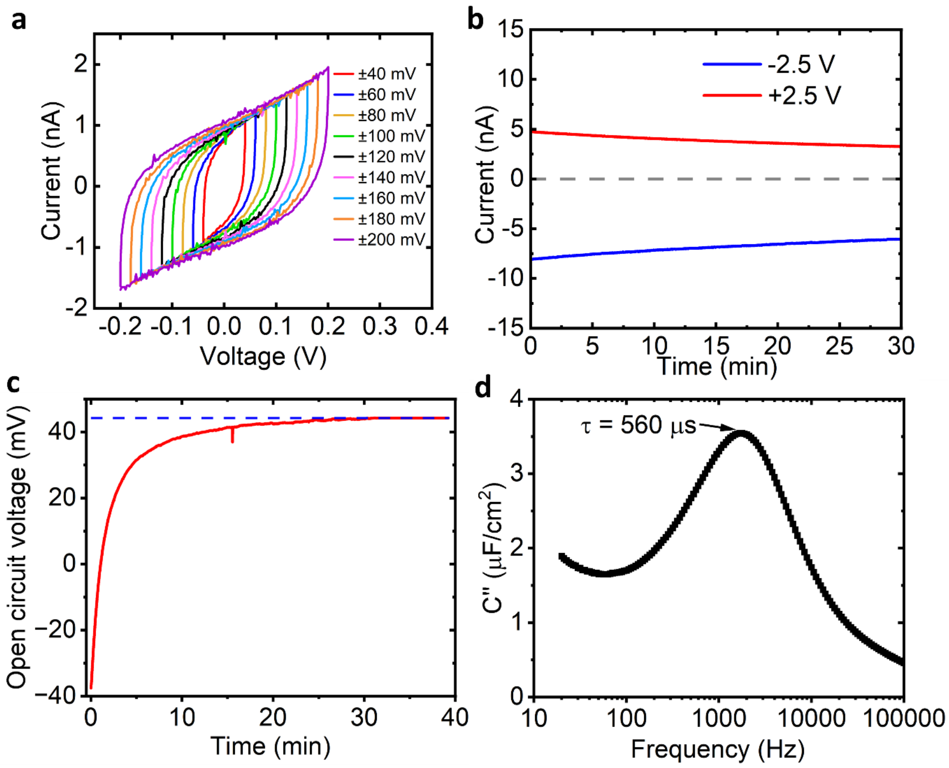

Figure S1. (a) Cyclic voltammograms for small voltage ranges. (b) Leakage current as a function of time for ±2.5 V. (c) Open circuit voltage as a function of time. (d) Imaginary part of the junction capacitance as a function of frequency, derived from electrical impedance spectroscopy measurements with 100 mV applied ac voltage.

Extraction of magnetic parameters

We used a 2 nm Ta/4 nm Pt/0.9 nm CoFeB (40:40:20)/0.2 nm Pt/10 nm LiPON/5 nm Pt thin-film heterostructure to extract the magnetic parameters. The sample showed a similar square polar MOKE hysteresis loop as the patterned supercapacitors under a negative bias voltage (Figure S2a). To extract the saturation magnetization (M_s) and the perpendicular magnetic anisotropy (K_u), we conducted vibrating sample magnetometry (VSM) measurements using an in-plane magnetic field (Figure S2b). From the data, we found $M_s = 1.20 \times 10^6$ A/m and $K_u = 9.96 \times 10^5$ J/m^3. The exchange constant was determined from the temperature dependence of the saturation magnetization, as shown in Figure S2c, using Eq. 6 in Ref. S2. This procedure gave $A_{ex} = 14$ pJ/m. Finally, to extract the Dzyaloshinskii-Moriya interaction constant (D), we demagnetized the sample and imaged the stripe domains at zero magnetic field (Figure S2d). We subsequently used the derived stripe period of 2.25 μm in micromagnetic simulations to extract D. For the micromagnetic simulations we utilized the MuMax3 package [S3, S4]. The simulations were performed on a 16348 × 512 × 1 grid with a 2.5 nm × 2.5 nm × 0.9 nm cell size. As input parameters we used the values of M_s, K_u and A_{ex} as given above. Stripe domains of different widths were initialized along the x-direction of the simulation. The D value which led to stripe domains

with a period of 2.25 μm corresponded to D = 0.74 mJ/m^2. This D value is significantly above the value required for the stabilization of Néel domain walls in the CoFeB layer. From the above parameters this value can be calculated using Eq. 23 in Ref. S5, which gives 0.22 mJ/m^2. This would mean that the bubble domains seen in the devices are skyrmions. To test the robustness of this finding we tested deviations from the experimentally obtained parameters. We find that a reduction of A_{ex} by 20% to 11.2 pJ/m leads to an extracted D = 0.58 mJ/m^2 and increasing M_s, with K_{eff} kept constant, by 20% to 1450 kA/m leads to D = 0.44 mJ/m^2, both of which are above the D value needed for Néel domain walls. We therefore conclude that significant errors in the experimental magnetic parameters would still lead to skyrmion bubbles in the CoFeB film.

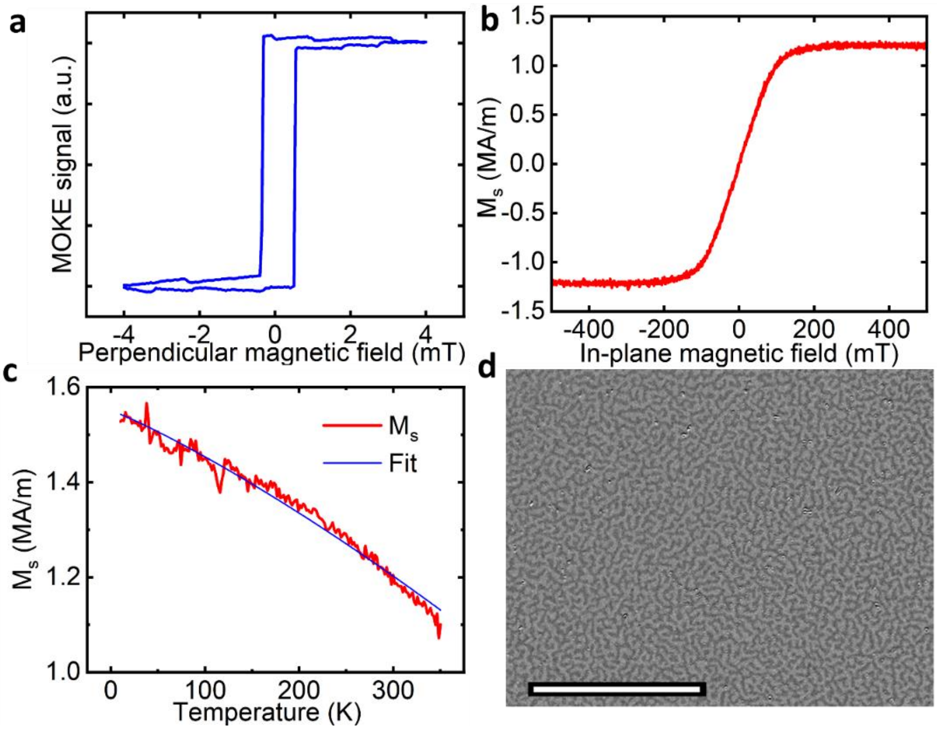

Figure S2. (a) Polar MOKE hysteresis loop of a 2 nm Ta/4 nm Pt/0.9 nm CoFeB (40:40:20)/0.2 nm Pt/10 nm LiPON/5 nm Pt thin-film heterostructure. (b) VSM magnetization curve taken under an in-plane magnetic field. (c) Saturation magnetization measured under 500 mT in-plane field as a function of temperature. The data is fitted to the modified Bloch law (see Ref. S2). (d) Magnetic stripe domains imaged by MOKE microscopy after sample demagnetization. The scale bar corresponds to 100 μm.

Micromagnetic simulations of the skyrmion energy

Figure S3 shows the simulated energy of the skyrmion state as a function of the skyrmion radius for different values of K_u and D. The simulations were carried out in MuMax3 software using a 2048 × 2048 × 1 grid with a 2.5 nm × 2.5 nm × 0.9 nm cell size. The saturation magnetization and exchange constant are fixed to $M_s = 1.20 \times 10^6$ A/m and A_{ex} = 14 pJ/m. For each combination of K_u and D,

skyrmions with different radii nucleate and relax. Figure S3 shows the skyrmion energy after relaxation as a function of skyrmion diameter. The black curve is simulated for the experimentally derived magnetic parameters of the thin-film heterostructure. Consistent with the lack of skyrmions in the as-grown sample (before the application of voltage), the minimum energy associated with a skyrmion having a diameter of around 1.2 μm is positive. Decreasing the perpendicular magnetic anisotropy by 1% (K_u = 9.86 × 10^5 J/m^3, D = 0.74 mJ/m^2) gives the orange curve, which shows a lower energy minimum and an expected skyrmion diameter of 1.2 – 1.4 μm. Decreasing the anisotropy further (K_u = 9.83 × 10^5 J/m^3, D = 0.74 mJ/m^2) stabilizes the skyrmions further (purple curve), while their diameter increases slightly to about 1.4 μm. A reduction of D (green and orange curves) increases the skyrmion energy and decreases the skyrmion size. The simulated skyrmion diameter of 1.2 – 1.4 μm compares well to the experimental observations (Figure 2c in the main paper).

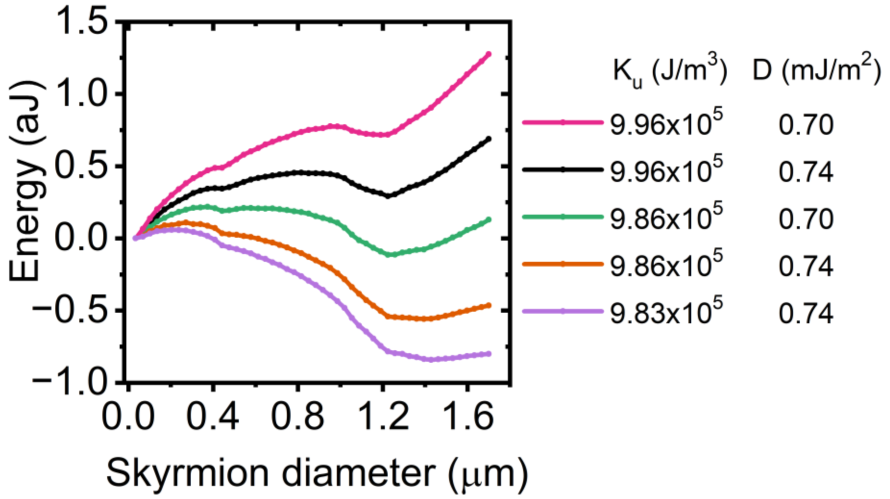

Figure S3. Simulated skyrmion energy as a function of skyrmion radius for different values of K_u and D. M_s = 1.20 × 10^6 A/m and A_{ex} = 14 pJ/m. The energy of the smallest skyrmion is set to zero.

Evolution of the skyrmion density after a voltage step

In Figure S4 the skyrmion density following a voltage step from 0 V to +2 V is shown with an applied magnetic field of 0.65 mT. The system reaches a steady-state after around 30 s. This is a response to both the changing Li content at the magnetic interface and the skyrmion nucleation dynamics. In this case, the applied voltage initially nucleates stripe domains which then transform into skyrmions. The timescale is a combination of the equilibration of the Li ion concentration and the equilibration of the magnetic state in response. When the voltage is switched to –2 V the skyrmions rapidly annihilate, as opposed to the situation where the voltage is not set negative but turned off, as in Figure 3a of the main paper and a slow decay of the skyrmion density occurs.

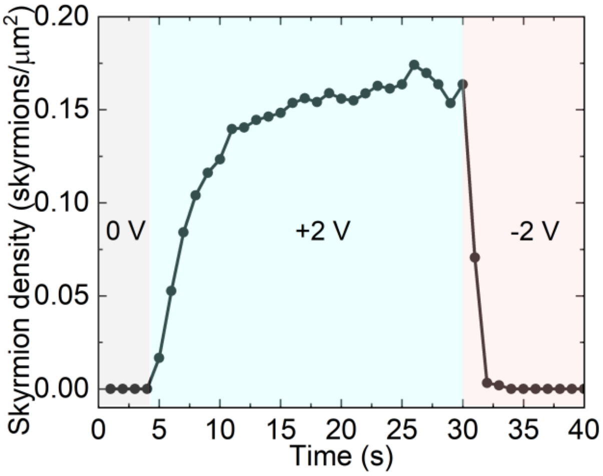

Figure S4. Response of skyrmion density with time during a voltage step, as labelled on the graph. A perpendicular magnetic field of 0.65 mT is applied during the whole period.

Fast voltage pulsing experiments

We recorded the evolution of the skyrmion density following short voltage pulses using MOKE microscopy. Figure S5 shows results for +10 V pulses with a duration of 150 μs and 200 μs. The positive voltage drives the Li ions towards the CoFeB/Pt interface, thereby lowering the energy of skyrmion nucleation. The accumulation of Li ions at the CoFeB/Pt interface peaks at the end of the 150/200 μs voltage pulse, however the skyrmion response is stretched over a longer time. The majority of the skyrmions nucleate on a timescale faster than our measurement, but it takes around 1 s for the skyrmion density to reach its maximum value. The slower magnetic response is explained by the thermally-activated nature of skyrmion nucleation. The Li ions slowly diffuse back to their equilibrium state within the LiPON electrolyte after the voltage pulse, which gradually reduces the number of skyrmions (see also Figure 3a in the main paper).

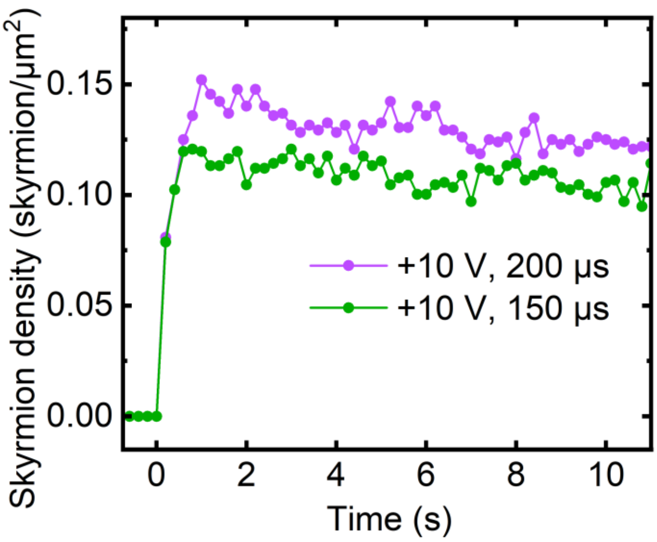

Figure S5. Time-evolution of the skyrmion density after applying +10 V pulses with a duration of 150 μs and 200 μs.

References

[S1] Tinevez, J.-Y.; Perry, N.; Schindelin, J.; Hoopes, G. M.; Reynolds, G. D.; Laplantine, E.; Bednarek, S.Y.; Shorte, S. L.; Eliceiri, K. W. TrackMate: An open and extensible platform for single-particle tracking. *Methods* **2017**, *115*, 80–90.

[S2] Zhou, Y.; Mansell, R.; Valencia, S.; Kronast, F.; van Dijken, S. Temperature dependence of the Dzyaloshinskii-Moriya interaction in ultrathin films. *Phys. Rev. B* **2020**, *101*, 054433.

[S3] Vansteenkiste, A.; Leliaert, J.; Dvornik, M.; Helsen, M.; Garcia-Sanchez, F.; Van Waeyenberge, B. The design and verification of MuMax3. *AIP Adv.* **2014**, *4*, 107133.

[S4] Mulkers, J.; Van Waeyenberge, B.; Milošević, M. V. Effects of spatially-engineered Dzyaloshinskii-Moriya interaction in ferromagnetic films. *Phys. Rev. B* **2017**, *95*, 144401.

[S5] Lemesh, I.; Büttner, F.; Beach, G. S. D. Accurate model of the stripe domain phase of perpendicularly magnetized multilayers. *Phys. Rev. B* **2017**, *95*, 174423.
